# Overexpression and Selectively Regulatory Roles of IL-23/IL-17 Axis in the Lesions of Oral Lichen Planus

**DOI:** 10.1155/2014/701094

**Published:** 2014-07-08

**Authors:** Rui Lu, Xin Zeng, Qi Han, Mu Lin, Long Long, Hongxia Dan, Gang Zhou, Qianming Chen

**Affiliations:** ^1^State Key Laboratory of Oral Diseases, West China College of Stomatology, Sichuan University, No. 14, Section 3, Renminnan Road, Chengdu 610041, China; ^2^The State Key Laboratory Breeding Base of Basic Science of Stomatology (Hubei-MOST) and Key Laboratory of Oral Biomedicine Ministry of Education, School and Hospital of Stomatology, Wuhan University, No. 237 Luoyu Road, Wuhan 430079, China

## Abstract

Interleukin- (IL-) 23/IL-17 axis is a newly discovered proinflammatory signaling pathway and has been implicated in the pathogenesis of many chronic inflammatory and immune disorders. Here we investigated whether the IL-23/IL-17 axis was present and functional in the lesions of oral lichen planus (OLP), a chronic inflammatory disease affecting the oral mucosa. Using immunohistochemistry and quantitative PCR, we found that the subunits of IL-23 and IL-17 were overexpressed in OLP lesions than in normal oral mucosa tissues. In addition, the expressions of IL-23 and IL-17 are positively correlated in reticular OLP tissues. Results from in vitro studies revealed that exogenous IL-23 could increase the percentage of Th17 cells and IL-17 production in the CD4+T cells from reticular OLP patients. Furthermore, we also found that exogenous IL-17 could significantly enhance the mRNA expressions of *β*-defensin-2, -3, CCL-20, IL-8, and TNF-*α*, but not *β*-defensin-1, CXCL-9, -10, -11, CCL-5, and IL-6 in human oral keratinocytes. Taken together, our results revealed an overexpression pattern and selectively regulatory roles of IL-23/IL-17 axis in the OLP lesions, suggesting that it may be a pivotal regulatory pathway in the complex immune network of OLP lesions.

## 1. Introduction

Oral lichen planus (OLP) is a chronic inflammatory disease affecting the oral mucosa, with a prevalence of about 1~2% of the population [1, 2]. Once established, the lesions rarely undergo self-remission and, in some cases, have a malignant potency [3]. Histologically, OLP is characterized by a dense subepithelial infiltration of lymphocytes, increased numbers of intraepithelial lymphocytes, and the degeneration of basal keratinocytes. The lymphocytic infiltrate consists predominantly of T cells [2, 4, 5].

Although the etiology of OLP remains unclear, accumulating evidence supports a role of immune dysregulation in the pathogenesis of OLP, especially involving a T-cell-mediated immune response and the abnormal production of various inflammatory molecules [2, 4, 6]. Previous studies have identified that a variety of proinflammatory molecules are aberrantly synthesized and secreted by both infiltrated T cells and altered keratinocytes in the lesions of OLP, which contribute to the onset and persistence of the inflammatory responses [7–12]. These findings indicated that OLP lesion development is closely associated with the activation, production, and functions of these inflammatory molecules.

The interleukin- (IL-) 23/IL-17 axis is a newly discovered proinflammatory signaling pathway, in which IL-23 and IL-17 are two pivotal cytokines [13, 14]. IL-23 is a heterodimeric proinflammatory cytokine, which is composed of a unique p19 subunit and a common p40 subunit shared with IL-12 [15]. Secreted by various cell types, such as activated dentritic cells, macrophages, and epithelium, IL-23 functions as an important driving factor in the immune response [15]. Notably, IL-23 serves an essential role in induction and maintenance of a novel subset of CD4+Th cells, namely, Th17 [16, 17]. In addition, IL-23 also induces Th17 to produce its distinctive cytokine IL-17, which is composed of two IL-17A subunits [18, 19]. Subsequently, IL-17 functions as a proinflammatory cytokine, which can activate different cells such as epithelial, endothelial, fibroblast, chondrocyte, and osteoblast to produce numerous inflammatory molecules including cytokines, chemokines, defensins, and MMPs [20, 21]. Together with these downstream proinflammatory products, the IL-23/IL-17 axis is extensively involved in the processes of chronic inflammation within various pathological states [20]. The IL-23/IL-17 axis has recently been described to play a major role in the pathogenesis of different chronic inflammatory and immune diseases, such as rheumatoid arthritis (RA), psoriasis, atopic dermatitis, inflammatory bowel disease (IBD), and periodontitis [22–29].

In our previous study, we have detected the serum and saliva levels of IL-17 in OLP patients but found no significant difference compared to healthy groups [30]. In others' investigation, the presence of IL-17 has been found in the lesions of OLP, suggesting its role in the local environments [31]. However, till now, the role of IL-23/IL-17 axis in the pathogenesis of OLP is still unclear. The aim of the present study was to investigate the expression patterns and regulatory roles of IL-23/IL-17 axis in OLP.

## 2. Materials and Methods

### 2.1. Patients, Controls, and Samples

All tissue and blood specimens of OLP patients and healthy volunteers were obtained from West China School and Hospital of Stomatology, Sichuan University. The OLP cases were clinically diagnosed, pathologically confirmed, and subdivided as reticular or erosive forms based on the modified WHO diagnostic criteria of OLP [32]. The two OLP subgroups were matched for age and sex. In addition, age and sex matched healthy individuals were recruited as control subjects. Subjects with other oral or systematic diseases or taking corticosteroids or immunosuppressive drugs within 3 months prior to the specimen collection were excluded from the recruitment.

For immunohistochemistry (IHC) staining, 27 OLP lesion specimens, including 13 with erosive form and 14 with reticular form, were recruited from the archives of the department of pathology, and 10 normal oral mucosa (NOM) tissues were collected from healthy volunteers receiving orthognathic surgery. For quantitative PCR analyses, 14 reticular OLP and 10 NOM tissues were obtained during the biopsy or orthognathic surgery, respectively, and subsequently snap-frozen in liquid nitrogen for the following experiment procedure. For studying the effect of IL-23 on the CD4+T cells, peripheral blood samples were collected from 10 of 14 reticular OLP patients noted above. The clinical characteristics of these participants were listed in Table 1.

Written informed consent was obtained from each subject, and the whole experiment procedure was conducted in accordance with the Declaration of Helsinki and approved by the Scientific and Ethical Committee Board of Sichuan University.

### 2.2. Immunohistochemistry

Paraffin-embedded sections of OLP lesion specimens and NOM tissues were treated in xylene and hydrated in graded ethanol, followed by blocking the activity of endogenous peroxidase with 3% hydrogen peroxide. After antigen retrieval by heat and press, the sections were incubated with goat anti-human IL-17 monoclonal antibody (1 : 100, R&D Systems, USA) or rabbit anti-human IL-23p19 monoclonal antibody (1 : 100, Abcam, UK) overnight at 4°C. Subsequently, sections were incubated with the rabbit anti-goat lgG antibody-HRP polymer or goat anti-rabbit lgG antibody-HRP polymer detection reagent (ZSGB Biotechnology, Beijing, China) for 15 minutes at room temperature and then 3,3′-diaminobenzidine tetrahydrochloride (DAB) for 1-2 minutes. After that, the sections were counterstained with hematoxylin. As negative controls, the nonimmune serum was used instead of the primary antibodies.

Evaluation of immunostaining was preformed independently by 2 observers in a blinded manner. The expression of IL-23p19 subunit was assessed using an arbitrary scoring system as follows: 0, no staining; 1, very weak staining (1–5 cells per section); 2, weak staining (5–30 cells per section); 3, moderate staining (30–100 cells per section); 4, strong staining (100–400 cells per section); and 5, strong staining (>400 cells per section). The average score in each group was calculated and compared. The expression activities were assessed as previously described [33, 34]. To evaluate the expression of IL-17, five high power fields (hpf) at a magnification of ×400 were randomly picked up and the numbers of IL-17+ cells were counted separately. The average number of IL-17+ cells per field was then calculated and compared.

### 2.3. Isolation and Culture of Peripheral Blood CD4+Th Cells

Five milliliters of heparinized blood was obtained from 10 OLP patients, respectively. The peripheral blood mononuclear cells (PBMCs) were isolated by Ficoll-Hypaque density-gradient centrifugation. Subsequently, CD4+T helper (Th) cells were purified by anti-human CD4 magnetic particles (BD biosciences, USA) on a cell separation magnet (BD Biosciences, USA) according to the manufacturer's instructions. The purified CD4+Th cells were resuspended at a density of 1 × 10^6^ cells/mL RPML 1640 medium (Thermo Scientific HyClone, Beijing, China) supplemented with 10% fetal bovine serum (FBS; GIBCO, Grand Island, NY, USA). For activation of CD4+Th cells, 10 *μ*g/mL of purified mouse anti-human CD3 antibody (BD Pharmingen, USA) and 5 *μ*g/mL of purified mouse anti-human CD28 antibody (BD Pharmingen, USA) were added. To study the effect of IL-23 on the CD4+Th cells, the cells were cultured with or without recombinant human IL-23 (rIL-23, 20 ng/mL; R&D Systems, Minneapolis, MN, USA) for 36 hours. Subsequently, cells and culture supernatant were collected separately for the following intracellular cytokine staining and ELISA detection.

### 2.4. Intracellular Cytokine Staining and Flow Cytometry

For intracellular cytokine staining, collected peripheral blood CD4+Th cells were resuspended at a density of 1 × 10^6^ cells/mL and stimulated in RPML 1640 medium containing phorbol myristate acetate ((PMA), 50 ng/mL, Sigma), ionomycin (1 *μ*g/mL, Sigma), and monensin (0.67 *μ*g/mL, Goldistop, BD Pharmingen, USA) for 4 hours. After that, cells were stained using a Cytofix/Cytoperm Fixation/Permeabilization Solution Kit (BD Pharmingen, USA), anti-human CD4 PERCP-CY5.5 antibody (BD Pharmingen, USA), and anti-human IL-17 APE antibody (BD Pharmingen, USA), successively, according to the manufacturer's instructions. Stained cells were analyzed using the FACSAria flow cytometer and BD FACSDiva software (BD Biosciences, San Diego, CA, USA).

### 2.5. ELISA

The concentration of IL-17 in the culture supernatants of peripheral blood CD4+Th cells was measured using a human IL-17 ELISA kit (BOSTER, Wuhan, China). According to the manufacturer's instructions, the detectable range of IL-17 content was from 31.2 to 2000 pg/mL.

### 2.6. Culture and Stimulation of Keratinocyte

HOK16E6E7, a human immortalized oral keratinocyte cell line, was plated in 6-well plates (2 × 10^5^/well) in keratinocyte serum-free medium (KSFM, Gibco BRL Life Technologies, Grand Island, NY, USA) containing supplemented with epidermal growth factor and calcium. After 24 hours' growth, medium with different doses of recombinant human IL-17 (rhIL-17, 0–20 ng/mL; R&D Systems, Minneapolis, MN, USA) or medium only was placed. After another 24 hours' culture, the cells were collected and kept in −70°C until the RNA isolation.

### 2.7. Real-Time Quantitative PCR

Total RNA was isolated from 14 reticular OLP and 10 NOM tissue specimens or the cultured cells using TRIzol reagent (Invitrogen, Carlsbad, CA, USA). cDNA was synthesized from 400 ng of RNA using the PrimeScript RT reagent Kit (TAKARA Biotechnology, Dalian, China). Primers purchased from TAKARA Biotechnology were listed in Table 2. To avoid the amplification of genome DNA, all the primers were exon-exon junction-spanning designed. The real-time quantitative PCR on 10 ng of cDNA was performed using the SYBR green detection assay on an Applied Biosystems 7300 Real-Time PCR System. The amplification procedure consisted of 10 s at 95°C, followed by 40 cycles of the amplification procedure composed of 95°C for 5 s and 62°C for 40 s. Each sample was run in triplicate. The relative gene expression normalized to the expression of GAPDH housekeeping gene and control sample were analyzed by the –ΔΔC_t_ method.

### 2.8. Statistical Analysis

All statistical analysis was performed on GraphPad Prism 5 software. Kruskal-Wallis test and Mann-Whitney test were used to determine differences between groups, paired *t*-test was used for the pair comparison, and Spearman's test was used to analyze the correlations. A value of *P* < 0.05 was considered as statistically significant.

## 3. Results

### 3.1. IL-23 and IL-17 Are Overexpressed in OLP Lesions

**Figure 1:**
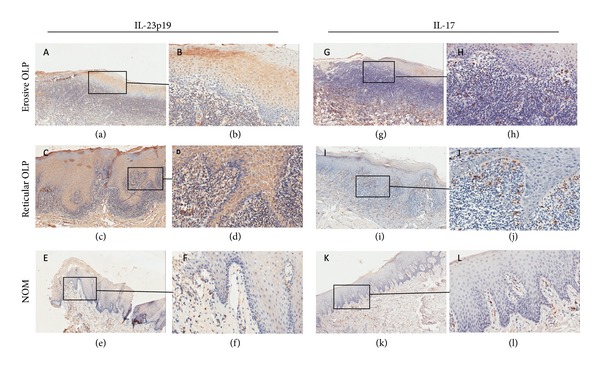
Immunohistochemical stainings for IL-23p19 (a–f) and IL-17 (g–l) in erosive (a, b, g, and h) and reticular (c, d, i, and j) OLP lesions and normal oral mucosa tissues (e, f, k, and l). Immunohistochemical staining for IL-23p19 showed diffuse and strong patterns in epithelium and the extracellular matrix of the lamina propria of both erosive ((a) ×100; (b) ×400) and reticular ((c) ×100; (d) ×400) OLP lesions, but weak or absent pattern in normal oral mucosa tissues ((e) ×100; (f) ×400). Abundant IL-17 positive staining was observed on the cytoplasm of the infiltrated lymphocytes in the lesions of both erosive ((g) ×100; (h) ×400) and reticular ((i) ×100; (j) ×400) OLP, but only a few sporadic IL-17+ cells were seen in normal oral mucosa ((k) ×100; (l) ×400).

**Figure 2:**
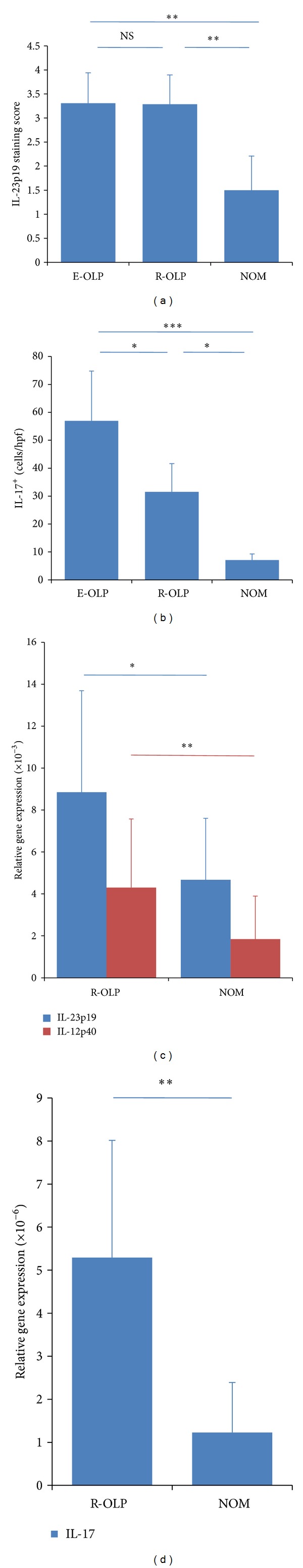
Expressions of IL-23 and IL-17 in OLP lesions. (a) The average staining scores of IL-23p19 in erosive OLP lesions (*n* = 13), reticular OLP lesions (*n* = 14), and normal oral mucosa tissues (*n* = 10). (b) The average number of IL-17+ cells per hpf in erosive OLP lesions (*n* = 13), reticular OLP lesions (*n* = 14), and normal oral mucosa tissues (*n* = 10). ((c) and (d)) The mRNA expressions of IL-23p19, IL-12p40, and IL-17 in reticular OLP lesions (*n* = 14) and normal oral mucosa tissues (*n* = 10). All data were shown as mean ± SEM. _ _**P < 0.01; _ _**P < 0.05; NS: nonsignificantly.

To identify whether IL-23/IL-17 is involved in the local pathogenesis of OLP, we firstly detected the expression and distribution of IL-23 p19, a unique subunit of IL-23, and IL-17 in OLP lesions and NOM tissues. Using IHC detection, we observed diffuse and strong expressions of IL-23p19 in both erosive and reticular OLP lesions. The positive staining of IL-23p19 predominantly concentrated on the epithelium of OLP lesions and also on the extracellular matrix of the lamina propria (Figures 1(a)–1(d)). In contrast, only a few keratinocytes in the epidermis layer of the NOM tissues showed weak stain of IL-23p19 (Figures 1(e) and 1(f)). Moreover, we found abundant IL-17 positive stainings on the cytoplasm of the infiltrated lymphocytes in the lesions of both erosive and reticular OLP, but only a few sporadic IL-17+ cells in the normal oral mucosa (Figures 1(g)–1(l)). The statistical data showed that both the erosive and reticular OLP lesions had significantly increased immunostaining scores of IL-23p19, as well as the numbers of IL-17+ cells, compared to the normal oral mucosa. In addition, erosive OLP lesions contained a significantly increased number of IL-17+ cells compared to the reticular OLP lesions. However, there is no significant difference in IL-23p19 staining score between erosive and the reticular OLP lesions (Figures 2(a) and 2(b)).

To verify the IHC results, we also detected the mRNA expressions of both subunits of IL-23 (IL-23p19 and IL-12p40) and IL-17 in 14 reticular OLP lesional tissues and 10 NOM tissues and found that the mRNA expressions of all the three genes in OLP lesions were significantly increased compared to NOM tissues (Figures 2(c) and 2(d)).

These data demonstrated overexpression of IL-23 and IL-17 in the OLP lesions, indicating that the IL-23/IL-17 axis may be involved in the local immune network of OLP.

### 3.2. The Expressions of IL-23 and IL-17 Are Positively Correlated in the Progress of OLP Lesions

**Figure 3:**
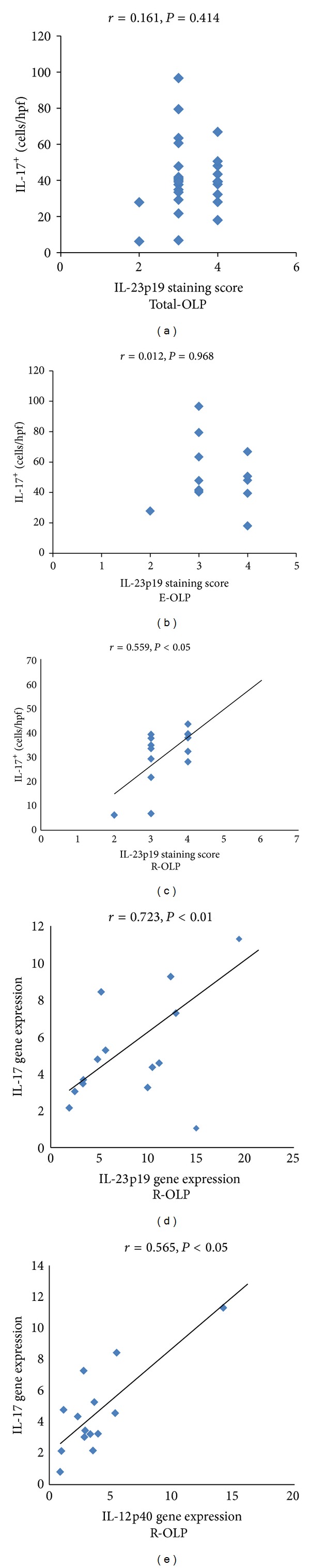
Correlation between the expressions of IL-23 and IL-17 in OLP tissue specimens. (a–c) Correlations between the staining scores of IL-23p19 and numbers of IL-17+ cells per hpf in total OLP tissue specimens (*n* = 27, *r* = 0.161, *P* > 0.05), erosive OLP tissue specimens (*n* = 13, *r* = 0.012, *P* > 0.05) and reticular OLP tissue specimens (*n* = 14, *r* = 0.559, *P* < 0.05). (d-e) Correlations between the mRNA expressions of IL-23p19 ((d) *r* = 0.723, *P* < 0.01) or IL-12p40 ((e) *r* = 0.565, *P* < 0.05) and IL-17 in reticular OLP tissue specimens (*n* = 14).

Considering IL-23 as an important upstream inducing cytokine of IL-17, we next investigated whether the upregulation of IL-23 in the progress of OLP lesion is associated with the increased expression of IL-17. Analyzing based on the data above, we found no correlation between the IL-23p19 staining scores and the numbers of IL-17+ cells in the OLP lesions (Figure 3(a)). However, in reticular OLP subgroup, there was a positive correlation between the IL-23p19 staining scores and the numbers of IL-17+ cells (Figure 3(c)), whereas no correlation was found in erosive OLP group (Figure 3(b)). Moreover, we also found that the mRNA expressions of both IL-23 subunits, IL-23p19 (Figure 3(d)) and IL-12p40 (Figure 3(e)), are positively correlated with mRNA expression of IL-17 in reticular OLP samples. These results showed that overexpressions of IL-23 and IL-17 are positively correlated in the reticular OLP lesion, indicating a potential regulatory role of IL-23 to the expression of IL-17 in the early stage of OLP lesion.

### 3.3. IL-23 Increases the Percentage of Th17 Cells and IL-17 Production in CD4+T Cells from OLP Patients

**Figure 4:**
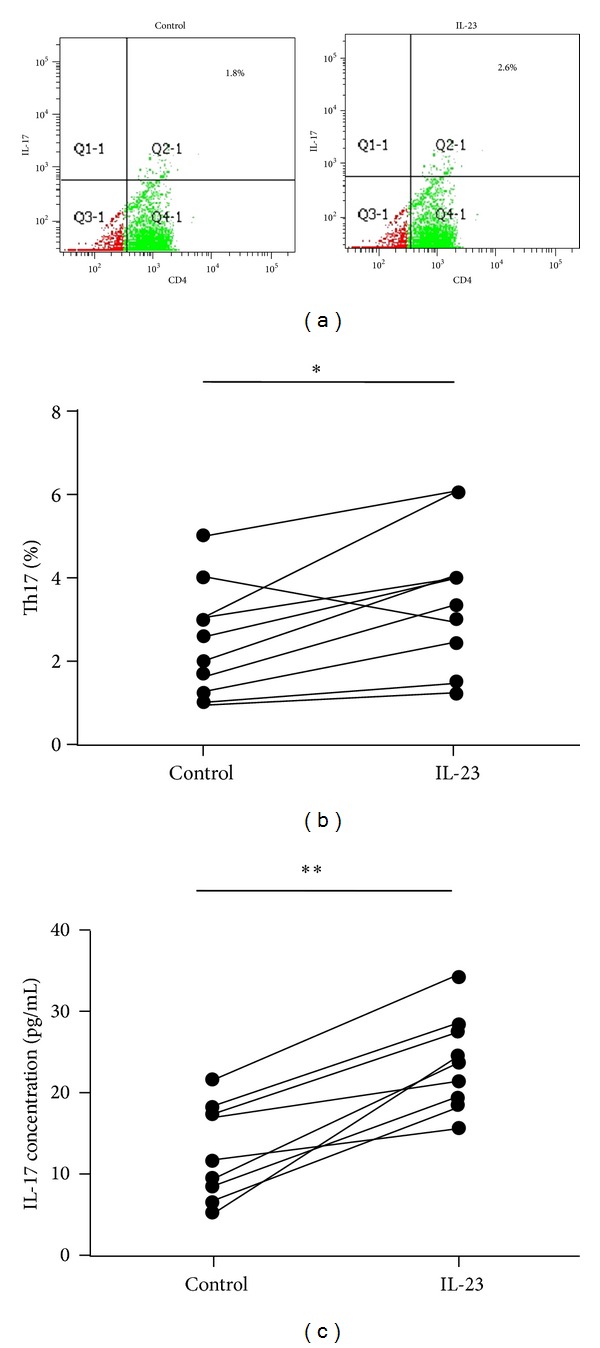
The effect of recombinant (r) IL-23 on the percentage of Th17 cells and IL-17 production in CD4+T cells from OLP patients. (a) Representative scatter plots of CD4+IL-17+ staining in peripheral blood CD4+T cells from OLP patients (*n* = 10), with or without the stimulation of rIL-23 (20 ng/mL) for 36 h. ((b) and (c)) Paired comparisons of percentages of Th17 cells (b) and the IL-17 content in the culture supernatant (c) in peripheral blood CD4+T cells from OLP patients (*n* = 10), with or without the stimulation of rIL-23 (20 ng/mL) for 36 h.

Next, we explored the potential role of IL-23 in the production of IL-17 in OLP. Although IL-17 has been recently reported to be produced by various cell types, a CD4+Th cell subset, namely, Th17 cell, is one of its main sources. On the other hand, in the local lesion of OLP, the dense subepithelial inflammatory infiltrate consists predominantly of CD4+Th cells. Therefore, here we focused on the effect of IL-23 to the IL-17 production in CD4+Th cells from 10 OLP patients. We observed that compared to the control group, the stimulation of rIL-23 significantly increased the percentage of CD4+IL-17+ cells (identified as Th17) in CD4+Th cells from OLP patients (Figures 4(a)–4(c)). In addition, the IL-17 content in the culture supernatant of CD4+Th cells also increased under the stimulation of IL-23 than the control group (Figure 4(d)). These data suggest that the overexpression of IL-23 in OLP lesions probably contributes to the induction of Th17 and the production of IL-17.

### 3.4. IL-17 Selectively Regulates the Expressions of Inflammatory Mediators in Oral Keratinocytes

We next explored the potential biological effects of IL-17, the essence effector of IL-23/IL-17 axis, in OLP lesions. It is well known that oral keratinocyte is an important component in the oral mucosa immunity and plays an important role in the pathogenesis of many chronic inflammatory oral diseases, including OLP, by producing various inflammatory mediators, such as cytokines, chemokines, and defensins. Therefore, we investigated the effect of IL-17 on the expressions of inflammatory mediators by HOK16E6E7, a human oral keratinocyte cell line.

**Figure 5:**
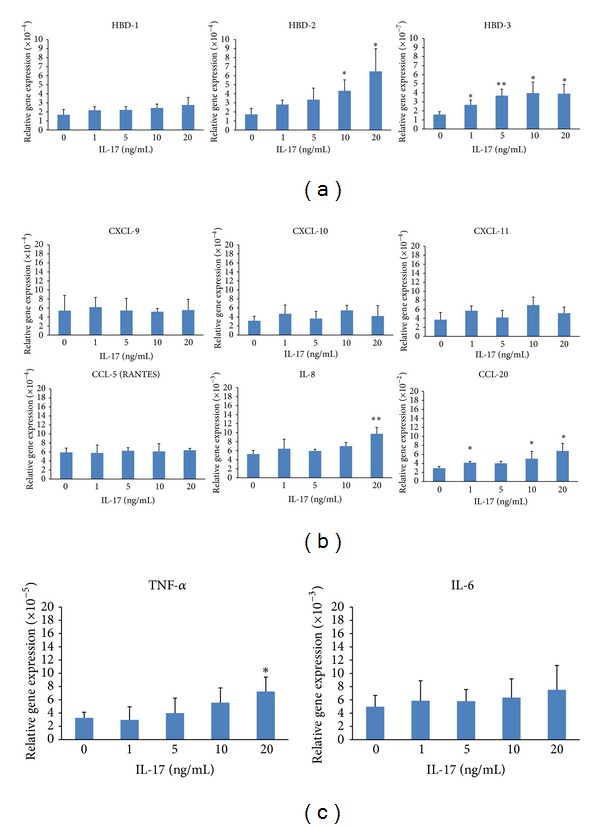
The effect of recombinant (r) IL-17 to the mRNA expressions of inflammatory mediators, including *β*-defensins (a), chemokines (b), and proinflammatory cytokines (c), on the HOK cells. Results were shown as mean ± SEM. _ _**P < 0.01; _ _**P < 0.05.

First we detected effect of IL-17 on the mRNA expression of human *β*-defensins (HBD) in HOK cells. We observed that IL-17 significantly increased mRNA expressions of HBD-2 and -3 in a dose-dependent manner, but not HBD-1 (Figure 5(a)). We next detected the mRNA expression of 6 chemokines and found that IL-17 could significantly increase the mRNA expressions of IL-8, CCL-20, but not CXCL-9, -10, -11 or CCL-5, in HOK cells (Figure 5(b)). Furthermore, we found that IL-17 significantly increased mRNA expressions of TNF-*α*, an important proinflammatory cytokine in the pathogenesis of OLP, in HOK. However, mRNA expression of IL-6 exhibited no significant difference with or without the effect of IL-17 (Figure 5(c)). The data revealed that the IL-17 could selectively regulate the expressions of some, but not all, inflammatory mediators in oral keratinocytes, indicating the selectively regulatory role of IL-23/IL-17 axis in the immune network in OLP lesions.

## 4. Discussion

In recent years, the IL-23/IL-17 axis has been widely described to play a pivotal role in the pathogenesis of different chronic inflammatory disorders [22]. However, study about the expression and regulatory role of this new axis in OLP is just at its beginning. In a recent study of our group, we found no difference in the serum levels of IL-23 and IL-17 between the OLP patients and controls [30]. The present study is focused on the expression and regulatory role of IL-23/IL-17 axis in the local environment of OLP lesions.

IL-23 is the upstream driving cytokine in the IL-23/IL-17 axis; its importance in inflammation and autoimmunity has been widely demonstrated [15]. To determine whether IL-23 is involved in the development of OLP, we first detected its expression in the OLP lesions compared with the NOM tissues. Structurally, IL-23 is composed of a unique p19 subunit and a common p40 subunit shared with IL-12. To avoid confusion, we only detected the immunostaining of IL-23p19 in the tissue specimens. However, in the quantitative PCR assay, we examined the mRNA expressions of both the IL-23p19 and IL-12p40, for the expression of each gene could influence the amount of the IL-23. Our results revealed an upregulation of IL-23p19 in both erosive and reticular OLP lesions compared to the NOM, indicating its involvement in the disease progress. As a driving factor of immune response, IL-23 is predominantly produced by the antigen presenting cells (APCs) including dentritic cells and macrophages, but its expression was also found in the keratinocytes from normal and psoriatic skin [34]. In addition, the epidermis in lesional psoriatic skin revealed markedly stronger IL-23p19 stain than the epidermis in normal skin [34]. Similarly, here we also observed a diffuse and strong expression pattern of IL-23p19 in the epithelial layer of OLP lesions, where keratinocytes are the major cell type. These findings suggested that keratinocytes may be a major source of IL-23 production under the chronic inflammatory condition in the mucocutaneous system. Although both the erosive and reticular OLP lesions had significantly increased immunostaining scores of IL-23p19 than the normal oral mucosa, the expressions of IL-23p19 between the erosive and reticular OLP lesions are similar. This observation suggests that the overexpression of IL-23 may be an early event in the pathogenesis of OLP lesion and be maintained at a high level in the later progress.

IL-17 is another key component in the IL-23/IL-17 axis and primarily functions as a downstream effector. Overexpressions of IL-17 have been observed in many autoimmune and inflammatory diseases, and its pivotal roles in the pathogenesis have been profoundly identified [20, 35]. Although IL-17 can be secreted by a variety of innate and adaptive immune cells, T cells are still its major sources, especially the new subset of CD4+Th cells, namely, Th17 [20]. Considering the involvement of a T-cell-mediated immune response in the pathogenesis of OLP, it is not surprising that IL-17 and Th17 cells may be present and play a regulatory role in the local environment of the disease. Indeed, we observed a large number of IL-17+ cells located in the subepithelial lymphocytic infiltrate in the OLP lesions. Besides, our data showed increased numbers of IL-17+ cells and higher mRNA expressions of IL-17 in the OLP lesions compared to the NOM tissues. In addition, compared to the reticular OLP lesions, erosive OLP lesions contained much more IL-17+ cells. These findings are consistent with the recent published study by Piccinni and his colleagues, who also found an elevated mRNA expression of IL-17, together with other Th17 type molecules in the OLP lesions compared to the healthy mucosa [36]. Moreover, the presence of Th17 was also identified in another recent study conducted by Xie et al. [31]. We must admit that the overexpression of IL-17 in OLP lesions is attributed partly to the infiltration of lymphocytes in the local environment. However, the large amount of IL-17 cytokine in the local lesions of OLP cannot be overlooked, for its potent proinflammatory properties may induce profound biological effects and play an important role in the formation and progress of the disease. On the other hand, Piccinni et al. also found that the CD4+T cell clones generated from OLP lesions produced significant higher levels of IL-17 than those generated from the healthy oral mucosa [36]. This phenomenon indicated that the overexpression of IL-17 in OLP lesions attributes not only to the lymphocytic infiltration, but also to other unknown regulatory mechanisms, which is needed to be further explored.

Since IL-23 is a major upstream inducer of IL-17 production, it is reasonable to further explore whether the upregulation of IL-23 has any regulatory role in the IL-17 production in the local environment of OLP. Based on the data, we conducted correlation analysis of the expressions of IL-23 and IL-17. Although no correlation between IL-23p19 staining scores and IL-17+ cell numbers was found in the total OLP group or erosive OLP subgroup, there were positive correlations between the expressions of IL-23 and IL-17 at both protein and mRNA levels in reticular OLP subgroup, indicating that the upregulation of IL-23 is associated with increased levels of IL-17 in the early stage of OLP lesion. On the other hand, the lacking of correlation between IL-23 and IL-17 expressions in erosive OLP lesions may be due to the persistent high levels of IL-23 but various levels of IL-17, indicating the existence of other potentially regulatory mechanisms, other than IL-23, in the IL-17 expressions in the erosive stage of OLP.

According to the recent published data by Xie et al., the IL-17 in the OLP lesion is mainly expressed in CD4+T cells, which is identified as Th17, in the subepithelial lymphocytic infiltration, as observations by double immunofluorescence staining [31]. In addition, CD4+T cell clones generated from OLP lesions exhibited an elevated activity in the IL-17 production [36]. Thus, we investigated the effect of IL-23 on the CD4+T cells from OLP patients. Our results showed that the stimulation of IL-23 could significantly increase the percentage of Th17 and the IL-17 production in CD4+T cells from OLP patients. Recent immunological findings have supported the opinion that, although IL-23 has no effect on the initiation of Th17 differentiation, it is crucially required for the proliferation and stability of precommitted Th17 cells, their further migration into the pathological tissues, and the production of IL-17, in which way to promote the pathogenic function of Th17 cells [37, 38]. Based on these findings and our data in the present study, it can be suggested that the overexpression of IL-23 may be functional and contributes, at least in part, to the accumulation of Th17 cells and the increased level of IL-17 in the OLP lesion. Therefore, the IL-23/IL-17 axis may represent a new signaling pathway in the crosstalk between the keratinocytes and CD4+T cells, which may be involved in the immunopathogenesis of OLP.

To investigate the potential biological effects of IL-23/IL17 in the OLP lesion, we further explored the effects of IL-17, the major effector of IL-23/IL-17 axis, on the production of different inflammatory mediators by the oral keratinocytes. Accumulated studies have demonstrated that oral keratinocyte is one of the major sources of the aberrant expressions of various inflammatory mediators in OLP lesions, including cytokines, chemokines, and defensins, which interact with each other and compose the complex immune network in the OLP environment [7–12, 39–41]. However, the mechanism of the functional alterations in the keratinocyte from OLP lesions is still unclear. Our results showed that the stimulation of IL-17 could significantly increase the mRNA expressions of HBD-2, 3, IL-8, CCL-20, and TNF-*α*, but not the expression of HBD-1, CXCL-9, -10, -11, CCL-5, and IL-6 in HOK cells. These data revealed that the effects of IL-17 on the oral keratinocyte are selective but not extensive, suggesting a unique regulatory role of IL-23/IL-17 axis in the local environment of OLP lesions.

**Figure 6:**
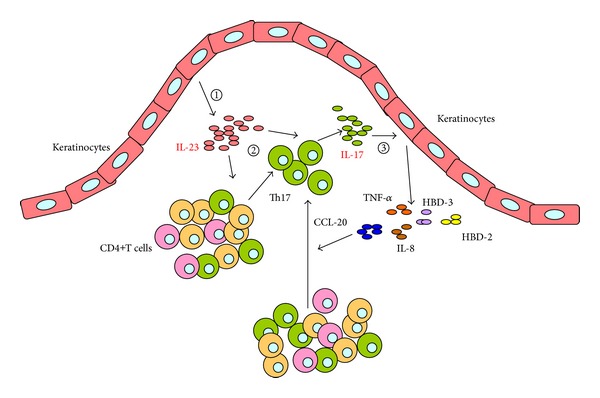
Schematic model of IL-23/IL-17 axis involved in the pathogenesis of OLP. The whole process is divided into three steps: (1) keratinocytes in OLP lesion produce a large amount of IL-23 via an unknown mechanism; (2) keratinocyte-derived IL-23 may contribute to the accumulation of Th17 cells and the overproduction of IL-17 in the local lesion of OLP; (3) IL-17 reversely induces the keratinocytes to selectively produce various inflammatory mediators, which compose the complex immune network in the inflammatory environment of OLP lesions.

In summary, based on the findings in the present study, we propose a novel model of interaction between the T cells and keratinocytes in the pathogenesis of OLP, in which the IL-23/IL-17 axis is involved (Figure 6). Firstly, keratinocytes in OLP lesion produce a large amount of IL-23 via an unknown mechanism. Next, keratinocyte-derived IL-23 may contribute to the accumulation of Th17 cells and the overproduction of IL-17 in the local lesion of OLP. Subsequently, IL-17 reversely induces the keratinocytes to selectively produce various inflammatory mediators, which compose the complex immune network in the inflammatory environment of OLP lesions. Our results warrant further explorations on the intrinsic mechanisms of the overexpression and the regulatory effects of IL-23/IL-17 axis, as well as its interaction with other signaling pathways, in the OLP lesions. Notably, IL-23/IL-17 axis has been recently considered as a relevant therapeutic target in chronic inflammatory and autoimmune diseases, and several biological agents blocking IL-23 or IL-17 have been currently developed [22, 42]. Therefore, further understanding of the role of IL-23/IL-17 axis in the pathogenesis may contribute to the development of novel therapeutic strategies for the prevention and management of OLP in the future.

## Figures and Tables

**Table 1:** Clinical features of the subjects.

	OLP		Control
	Erosive form	Reticular form	
Number	13	14	10
Gender			
Male	3	3	2
Female	10	11	8
Age (year)			
Range	23~52	26~49	20~48
Mean ± SD	43.9 ± 11.3	42.3 ± 12.6	40.8 ± 15.3
Site of biopsy			
Buccal	11	12	10
Tongue	2	2	

**Table 2:** Primer design for real-time quantitative PCR.

Gene	GeneBank accession number	Primer sequences	PCR product size (bp)
IL-17	NM_002190.2	Forward: 5′-atgactcctgggaagacctcat-3′	150
		Reverse: 5′-gttcaggttgaccatcacagtc-3′	

IL-23p19	NM_016584.2	Forward: 5′-ccttctctgctccctgatagc-3′	118
		Reverse: 5′-gactgaggcttggaatctgct-3′	

IL12p40	NM_002187.2	Forward: 5′-ctggagaaatggtggtcctca-3′	113
		Reverse: 5′-gacttggatggtcagggttttg-3′	

*β*-Defensin-1	NM_005218.3	Forward: 5′-ccttctgctgtttactctctgc-3′	126
		Reverse: 5′-gaatagagacattgccctccac-3′	

*β*-Defensin-2	NM_004942.2	Forward: 5′-gggtcttgtatctcctcttctcg-3′	130
		Reverse: 5′-ctagggcaaaagactggatgac-3′	

*β*-Defensin-3	NM_001081551.2	Forward: 5′-ccaggtcatggaggaatcat-3′	113
		Reverse: 5′-gagcacttgccgatctgttc-3′	

CXCL-9	NM_002416.1	Forward: 5′-gggactatccacctacaatcctt-3′	127
		Reverse: 5′-ctgctgaatctgggtttagacat-3′	

CXCL-10	NM_001565.3	Forward: 5′-gctgtacctgcatcagcattagt-3′	138
		Reverse: 5′-gacatctcttctcacccttctttt-3′	

CXCL-11	NM_005409.4	Forward: 5′-ttgtgtgctacagttgttcaagg-3′	110
		Reverse: 5′-atggaggctttctcaatatctgc-3′	

CCL-5	NM_002985.2	Forward: 5′-catattcctcggacaccacac-3′	131
		Reverse: 5′-ctttcgggtgacaaagacgac-3′	

CCL-20	NM_004591.2	Forward: 5′-tgtgctgtaccaagagtttgctc-3′	124
		Reverse: 5′-tgaagaatacggtctgtgtatccaa-3′	

IL-8	NM_000584.3	Forward: 5′-agctctgtgtgaaggtgcagtt-3′	126
		Reverse: 5′-ggtccactctcaatcactctcag-3′	

IL-6	NM_000600.3	Forward: 5′-ggagacttgcctggtgaaaatc-3′	140
		Reverse: 5′-gcaggaactggatcaggactt-3′	

TNF-*α*	NM_000594.3	Forward: 5′-aagcctgtagcccatgttgtag-3′	112
		Reverse: 5′-gctggttatctctcagctccac-3′	

GAPDH	NM_002046.4	Forward: 5′-ctttggtatcgtggaaggactc-3′	132
		Reverse: 5′-gtagaggcagggatgatgttct-3′	
