# Correction to “Overexpression and Selectively Regulatory Roles of IL‐23/IL‐17 Axis in the Lesions of Oral Lichen Planus”

**DOI:** 10.1155/mi/9805254

**Published:** 2026-04-25

R. Lu, X. Zeng, Q. Han, et al., “Overexpression and Selectively Regulatory Roles of IL‐23/IL‐17 Axis in the Lesions of Oral Lichen Planus.” *Mediators of Inflammation*. 2014 (2014): 701094, https://doi.org/10.1155/2014/701094.

After initial publication of the article, the authors identified an inadvertent error in Figure 4a, where the representative flow cytometry plot for CD4+IL‐17+ staining in peripheral blood CD4+ T cells from OLP patients stimulated with recombinant IL‐23 (right panel) was erroneously replaced with the image of the unstimulated control group (left panel) during the proof‐correction stage. The corrected Figure 4a is displayed below. This change does not affect the final conclusions.

**Figure 4:**
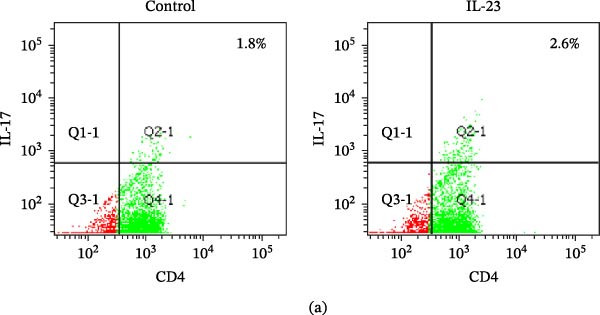
The effect of recombinant (r) IL‐23 on the percentage of Th17 cells and IL‐17 production in CD4+T cells from OLP patients. (a) Representative scatter plots of CD4+IL‐17+ staining in peripheral blood CD4+T cells from OLP patients (*n* = 10), with or without the stimulation of rIL‐23 (20 ng/mL) for 36 h. ((b) and (c)) Paired comparisons of percentages of Th17 cells (b) and the IL‐17 content in the culture supernatant (c) in peripheral blood CD4+T cells from OLP patients (*n* = 10), with or without the stimulation of rIL‐23 (20 ng/mL) for 36 h.

In addition, in paragraph 3.3 of the “Results” section, the citations in the text “(Figure 4a–c)” and “(Figure 4d)” were incorrect. These citations should be corrected to “(Figure 4a,b)” and “(Figure 4c),” respectively.

We apologize for these errors.

